# Design and Implementation of a Two-Wheeled Vehicle Safe Driving Evaluation System

**DOI:** 10.3390/s24144739

**Published:** 2024-07-22

**Authors:** Dongbeom Kim, Hyemin Kim, Suyun Lee, Qyoung Lee, Minwoo Lee, Jooyoung Lee, Chulmin Jun

**Affiliations:** 1 Department of Geoinformatics, University of Seoul, 163, Seoulsiripdae-ro, Dongdaemun-gu, Seoul 02504, Republic of Korea; dbkim@uos.ac.kr (D.K.); kimhm77@uos.ac.kr (H.K.); stbry27@uos.ac.kr (S.L.); 2 Technical Research Institute, Citus Co., Ltd., SJ Technoville, 278, Beotkkot-ro, Geumcheon-gu, Seoul 08511, Republic of Korea; qylee@citus.co.kr; 3 Technical Research Institute, Newns Co., Ltd., 66, Gunpocheomdansaneop 2-ro, Gunpo-si 15880, Republic of Korea; lmwsttt@naver.com; 4 Korea Gyeonggido Co., Ltd., 20, Pangyo-ro, Seongnam-si 13488, Republic of Korea; banzzak8@kgcbrand.com

**Keywords:** two-wheeled vehicles, driving behavior, safety evaluation system, risk factors, testbed, rider safety, mobile app

## Abstract

The delivery market in Republic of Korea has experienced significant growth, leading to a surge in motorcycle-related accidents. However, there is a lack of comprehensive data collection systems for motorcycle safety management. This study focused on designing and implementing a foundational data collection system to monitor and evaluate motorcycle driving behavior. To achieve this, eleven risky behaviors were defined, identified using image-based, GIS-based, and inertial-sensor-based methods. A motorcycle-mounted sensing device was installed to assess driving, with drivers reviewing their patterns through an app and all data monitored via a web interface. The system was applied and tested using a testbed. This study is significant as it successfully conducted foundational data collection for motorcycle safety management and designed and implemented a system for monitoring and evaluation.

## 1. Introduction

The rapid expansion of lifestyle logistics services necessitates proactive measures to address the safety concerns associated with two-wheeled vehicles. By 2022, the Republic of Korea delivery service market had surged to approximately KRW 26.5 trillion, marking remarkable growth of 973% compared to 2017, with an impressive annual growth rate of 162% (KOSTAT, http://www.kostat.go.kr, accessed on 1 June 2023). The shift towards non-face-to-face consumption patterns, a response to the COVID-19 pandemic, has led to the heightened utilization of two-wheeled vehicles, consequently resulting in an increase in traffic accidents related to deliveries. Of particular concern is the escalating prevalence of traffic accidents involving efficient two-wheeled vehicles, which are chosen for their accessibility and cost-efficiency. According to the Ministry of Land, Infrastructure, and Transport, as of 2022, the total number of traffic accident fatalities in Republic of Korea had decreased by 6.21% compared to the previous year, amounting to 2735 deaths [1]. However, fatalities involving two-wheeled vehicles increased by 5% to 484 deaths, emphasizing the urgency for enhanced safety measures dedicated to such vehicles.

In the context of vehicle safety management, there are broadly two perspectives: incentive-based approaches and penalty-based approaches. Incentive-based approaches encourage safe driving through economic rewards, promoting a culture of safe driving through voluntary participation. Incentive-based approaches leverage monetary rewards and direct feedback mechanisms, such as in-vehicle telematics, to foster safer driving behaviors and cultivate a culture of road safety among drivers [2,3,4]. However, they may involve initial costs. Penalty-based approaches maintain traffic safety through systems based on demerit points, providing immediate deterrence but requiring time for institutionalization. Penalty-based approaches employ immediate penalization for traffic violations to deter risky driving behaviors effectively and bolster comprehensive road safety initiatives [5]. Additionally, applying penalty-based approaches to motorcycles poses practical challenges due to the lack of existing monitoring systems, making it difficult for riders to attach the necessary sensors for compliance monitoring.

The data collection system and quantitative metrics employed for the evaluation of safe driving behaviors among operators of two-wheeled vehicles are found to be inadequate. In contrast, commercial vehicles such as buses and taxis benefit from well-defined criteria encompassing eleven categories of risky driving behaviors. These criteria are meticulously established through the analysis of passenger discomfort metrics, which are assessed using pupil reaction and electrocardiogram tests. Furthermore, a comprehensive safety driving index has been crafted, weighing specific violations, referred to as the Electronic Traffic Assessment Score (“eTAS”). In the realm of data collection systems for commercial vehicles, a plethora of real-time parameters, including the GPS coordinates, velocity, engine revolutions per minute (RPM), brake utilization, and traveled distance, are meticulously logged through the use of a digital tachograph (DTG). These data are then exploited to dissect and understand the vehicle operation patterns. Another driver safety monitoring service is the “TMAP Driving Habit Service”. Unlike the previously mentioned solutions for commercial vehicles, this service targets general users, specifically those driving four-wheeled vehicles. Based on driving data, the service can assess the driver’s habits and identify driving patterns to promote safer driving. Additionally, as a private service, it offers economic benefits such as insurance premium discounts linked to driving habits. In this way, Republic of Korea has various evaluation systems in place to encourage safe driving for both commercial vehicle operators and general four-wheeled vehicle users.

For two-wheeled vehicles, commercial services supporting safe driving are available in countries such as Germany and Israel. In Germany, the “Bosch Advanced Driver Assistance Systems” uses advanced cruise control (ACC) sensors to adjust the vehicle speed according to the traffic flow and maintain a safe following distance. If another vehicle is detected nearby and the rider does not react, the system issues warnings via auditory or visual signals. This system is based on a LiDAR-based motorcycle safety system, which directly manages safety through a combination of LiDAR sensors, brake systems, engine management, and a human–machine interface (HMI). In Israel, "RideVision" offers a customized advanced rider assistance system (ARAS) designed to meet the safety needs of riders. It operates through the coordination of two wide-angle cameras, a predictive vision algorithm, a compact embedded computer processor, and two mirror-mounted LED warnings. The app stores individual riding statistics and GPS tracking records, enabling riders to access data such as the mileage, location, incline, and speed, and provides summary reports based on this information. Thus, beyond merely monitoring the ride, there are systems in place that directly support rider safety.

However, in Republic of Korea, it is evident that the absence of dedicated data collection and analytical platforms tailored to two-wheeled vehicles has resulted in a substantial dearth of fundamental data. Two-wheeled vehicles, endowed with a higher degree of maneuverability compared to their four-wheeled counterparts, are prone to manifesting more risky driving behaviors, including rapid turns and impromptu lane changes. Consequently, the arsenal of methodologies for the detection and evaluation of aggressive driving conduct among two-wheeled vehicles remains notably constrained. This limitation has contributed to studies reliant on confined assessment criteria, which predominantly focus on attributes such as speed, acceleration, and the work environment [6]. These traditional approaches, however, fall short in the quest for quantitative indicators that holistically encompass the myriad driving behaviors exhibited by two-wheeled vehicles, particularly behaviors that can potentially precipitate traffic accidents, including signal violations, sidewalk encroachments, irregular lane changes, and retrograde driving. Therefore, it is necessary to establish a system for the collection of basic data before implementing systems that directly guide the safety of two-wheeled vehicles.

The development of a data-driven two-wheeled vehicle driving assessment system is imperative for several compelling reasons. First and foremost, the distinct and unrestrained mobility patterns exhibited by two-wheeled vehicles, such as motorcycles and scooters, necessitate the integration of specialized detection devices [7,8,9]. In the context of two-wheeled vehicle operation, distinguishing between roadways and sidewalks and employing GIS-based risk driving detection mandates the use of high-precision GPS devices. These devices provide the granular data required for a comprehensive assessment of two-wheeled vehicle operation. Existing pre-trained models and datasets, often based on four-wheeled vehicle black-box footage, face incompatibilities when applied to two-wheeled vehicles. The idiosyncrasies of two-wheeled vehicle behavior demand the creation of new datasets and the development of unique model weights specific to this mode of transportation [10,11]. This can be achieved through the implementation of on-board two-wheeled vehicle cameras. Moreover, ethical considerations make it impractical and unsafe to collect reference data for reckless driving directly from real two-wheeled vehicle riders. Consequently, there is a pressing need for the development and utilization of high-performance simulators capable of accurately reproducing real-world scenarios. Such simulators provide a means of constructing reference datasets for reckless driving under controlled conditions. These factors, when considered together, highlight the urgency of creating a comprehensive safety index tailored to two-wheeled vehicles. To this end, the development of a specialized data-driven two-wheeled vehicle driving assessment system becomes essential.

**Figure 1:**
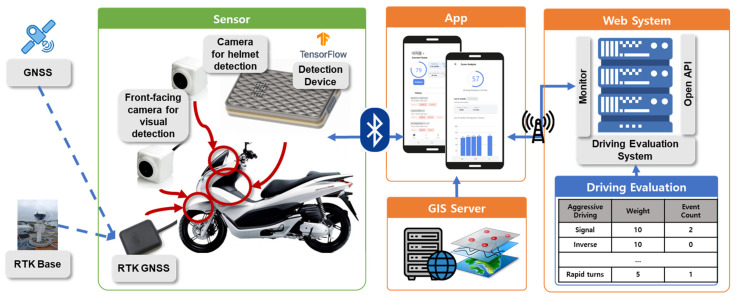
A schematic diagram of the two-wheeled vehicle monitoring system.

The primary objective of this study was to design and implement a monitoring system for two-wheeled vehicles to collect basic data. Subsequently, this system will be implemented and validated in a real-world testbed environment. To achieve these objectives, this study designed and implemented the system depicted in Figure 1. The system comprises physical sensors, an app and web system, and a driving assessment system. It verifies the precise locations of two-wheeled vehicles using GNSS and RTK and captures image-based visuals through front and rear cameras. It detects vehicle aggressiveness through detection devices. The collected data are transmitted to a smartphone app installed by the two-wheeled vehicle’s rider via BLE communication. The app monitors risk factors based on the rider’s location compared to a GIS server. Both the app and web system communicate via HTTPS, enabling the integrated monitoring and evaluation of the two-wheeled vehicle’s trajectories. Initially, the study defines the specific risk factors to be detected from two-wheeled vehicles (Section 2.1) and establishes detection methods for each factor (Section 2.2). Subsequently, the research delves into the intricacies of on-board detection devices designed for two-wheeled vehicles (Section 3.1) and provides an overview of the entire system (Section 3.2). Section 4 documents the empirical testing of the system in a real-world testbed area, and Section 5 concludes the study, summarizing its findings and implications.

## 2. Aggressive Driving of Two-Wheeled Vehicles

In this section, we define the risk factors associated with two-wheeled vehicles and discuss the methods for the identification of these factors on a per-item basis. Prior to the development of a system for the assessment of two-wheeled vehicle operation, it is essential to establish a clear definition of the specific factors to be evaluated. Subsequently, we provide a detailed description of the detection methods for each assessment factor.

### 2.1. Definition of Aggressive Driving

In Korea, similar technologies (KoROAD, https://www.koroad.or.kr/, accessed on 1 June 2023) have defined 11 risky driving behaviors for commercial vehicles such as trucks, buses, and taxis, which include speeding, extended speeding, rapid acceleration, sudden starts, abrupt deceleration, abrupt stops, quick lane changes, sudden overtaking, rapid left and right turns, and U turns. A study by the Korea Transport Safety Authority [1], released in January 2022, introduced a set of eight risky driving behaviors for two-wheeled vehicles, encompassing the speeding rate, frequency of rapid acceleration/deceleration, instances of abrupt lane changes/overtaking/turns, and incidents of falling and impact. A commercial service, “Riderlog”, developed by “Byulddaragaja” [6], evaluates drivers’ performance using motion sensors and GPS data. It assesses parameters such as the road traveled, shock history, rapid acceleration and deceleration, rolling, and sharp turns, culminating in a driving score.

**Figure 2:**
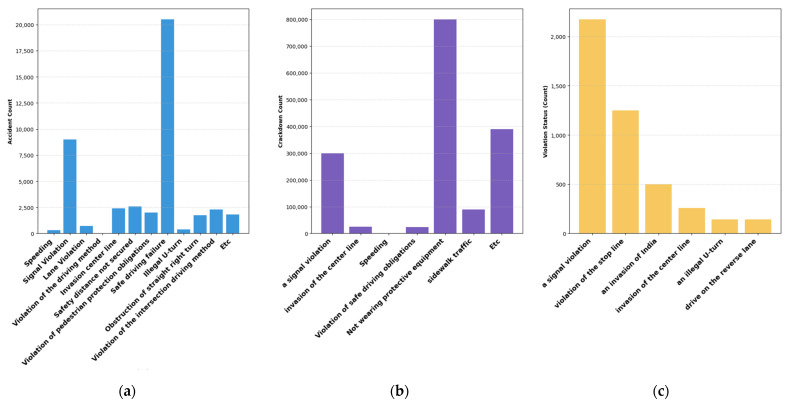
Chart of two-wheeled vehicle accidents and violations of laws and regulations. (**a**) The number of motorcycle accidents by violation of laws and regulations in the metropolitan area of Korea (Seoul, Incheon, Gyeonggi) between 2017 and 2021. (**b**) The status of crackdowns by motorcycle violations of laws and regulations between 2017 and 2021. (**c**) The result of the second fact-finding survey of two-wheeled vehicle traffic laws on 16 intersections in Seoul from 8 to 9 September 2021.

In this study, we derive 11 assessment criteria through three primary data sources: (1) the collection of two-wheeled vehicle traffic accident data over approximately five years using the TAAS Traffic Accident Analysis System, (2) the enforcement status of two-wheeled vehicle regulations based on data from the National Police Agency, and (3) data from the police database detailing two-wheeled vehicle regulation violations and corresponding traffic accident statistics. Key visualizations illustrating the types of two-wheeled vehicle traffic accidents from these data sources are presented in the following figures. Figure 2a displays the number of two-wheeled vehicle accidents by violation category in the capital region of Republic of Korea (Seoul, Incheon, Gyeonggi) from 2017 to 2021 (KoROAD, https://www.koroad.or.kr/, accessed on 1 June 2023). Among the various violation categories, non-compliance with safe driving rules accounts for the highest number of accidents, emphasizing the need for improved safety measures in two-wheeled vehicle operations. Figure 2b illustrates the enforcement status of two-wheeled vehicle regulations from 2017 to 2021, as reported by the National Police Agency. Notably, non-compliance with helmet usage ranks first in this category, highlighting the significant issue of helmet non-compliance among two-wheeled vehicle riders. Figure 2c presents the results of a secondary survey on two-wheeled vehicle regulation compliance conducted by KOSTA on 16 intersections along Seoul’s side streets during three hours on 8–9 September 2021. The statistics presented earlier indicate that two-wheeled vehicle riders commonly violate traffic signal regulations, resulting in frequent accidents. This underscores the need for assessment criteria that are distinct from those used for four-wheeled vehicles, considering the unique operational patterns of two-wheeled vehicles and the mixed objectives of delivery riders.

In this study, the evaluation criteria for two-wheeled vehicles were categorized into three main categories, traffic regulation violations, pedestrian endangerment, and aggressive driving, totaling 11 evaluation items (Table 1). The category of traffic regulation violations was further subdivided into five sub-items: signal violation, wrong-way driving, crossing the centerline, speeding, and helmet non-compliance. The pedestrian endangerment category included two sub-items: the proximity to pedestrians and riding on sidewalks. The aggressive driving category was defined by four items: rapid acceleration, abrupt deceleration, sudden turns, and swift lane changes.

### 2.2. Aggressive Driving Identification

The defined evaluation criteria for risky driving behaviors were categorized into three major detection methods: GNSS/GIS-based, image-based, and IMU sensor-based (Table 2). Signal violations, helmet non-compliance, proximity to pedestrians, and riding on sidewalks were evaluated using image-based detection methods. Wrong-way driving, crossing the centerline, speeding, and sidewalk violations were detected using high-precision GNSS/GIS-based methods. The category of aggressive driving was assessed using IMU sensor-based detection.

#### 2.2.1. Image-Based Identification

**Figure 3:**
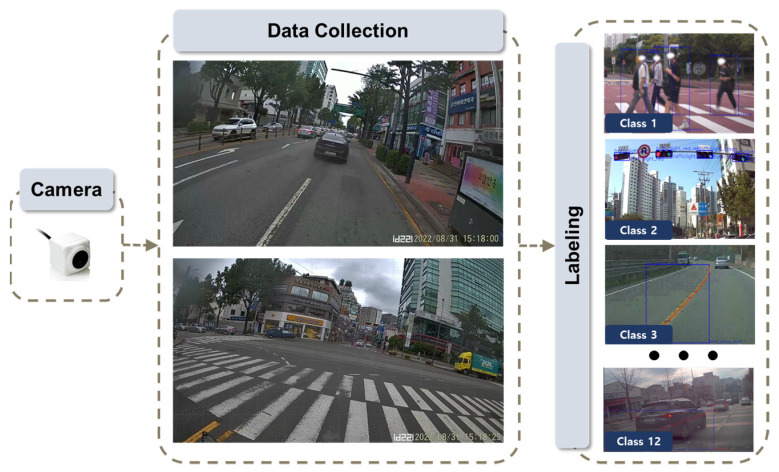
Flowchart to build an image-based reference.

To detect risky driving behaviors using an image-based approach, deep learning models were employed. Prior to this, the acquisition of reference and labeling data is of paramount importance. To detect instances such as signal violation, helmet non-usage, proximity to pedestrians, and riding on sidewalks, these image-based methods necessitate detection and subsequent classification. Therefore, it was imperative to secure both black-box footage from conventional four-wheel vehicles and footage from two-wheeled vehicles, which exhibit more freedom in their driving patterns. In this study, cameras were mounted at the front and rear of the two-wheeled vehicle to capture images. Figure 3 illustrates the process of data labeling. Following data collection, labeling work was undertaken to distinguish 12 different classes for the construction of the training dataset. These classes include lane and lane separation, traffic signal states, helmet usage by two-wheeled vehicle riders, and the identification of pedestrians.

#### 2.2.2. GIS-Based Identification

**Figure 4:**
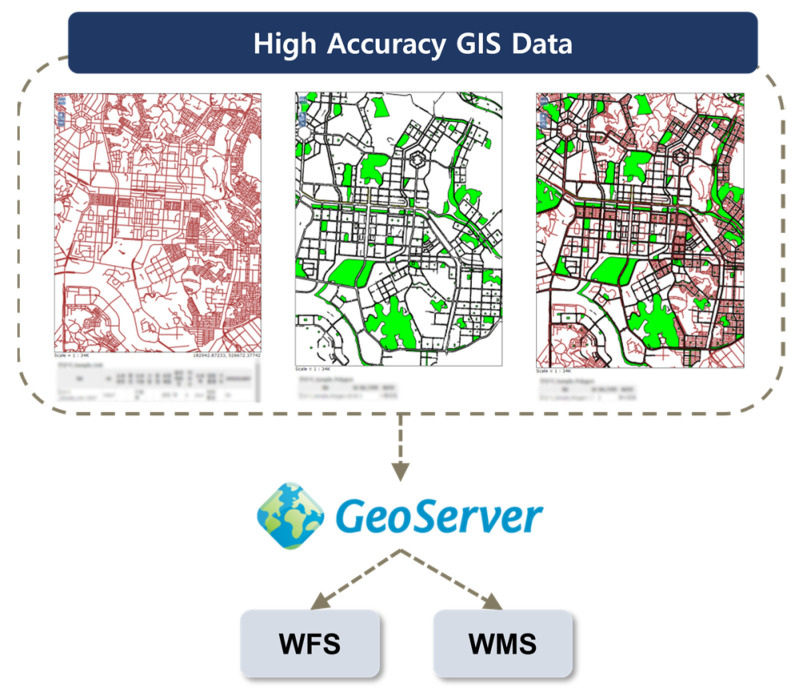
Flowchart to build a reference based on GIS/GNSS.

To detect risk factors based on GNSS/GIS data, high-precision GIS data were obtained. This dataset primarily consisted of spatial information pertaining to roads and buildings, with distinctions made down to the level of lanes and sidewalks to account for the free driving patterns of two-wheeled vehicles. High-precision GIS data were loaded into GeoServer, an open-source GIS middleware server developed in Java, and these reference data were made accessible to the detection models through the Web Feature Service (WFS) and Web Map Service (WMS), as depicted in Figure 4. GeoServer, being an open-source GIS software middleware server, facilitates the sharing and editing of GIS data [39]. Developed with interoperability in mind, it employs development standards to enable the service of various spatial data sources. In this study, the WFS was utilized to perform computations using detailed road network information, while the WMS allowed the provision of comprehensive road and building data to applications and the web. It is worth noting that the data used in this study were specifically constructed due to the limited availability of publicly accessible data.

#### 2.2.3. Inertial-Sensor-Based Identification

For sensor-based data, we have time series data from IMU sensors measuring the speed, gyro, and acceleration. The sensor-based risk factor in this study falls under the category of aggressive driving, which, unlike other evaluation criteria, lacks predetermined quantitative benchmarks. To address this, we established criteria through a web-based survey involving driving videos featuring aggressive driving scenarios simulated using a high-performance simulator, Carla.

The Carla simulator is an open-source platform primarily designed for autonomous driving research [40]. Beyond its applications in autonomous driving, it offers a valuable environment for the generation and modeling of trajectory data relevant to two-wheeled vehicles. This simulator virtually recreates various road conditions and environments, emulating the behavior of two-wheeled vehicles. It utilizes sensors, including speed, gyroscope, and acceleration sensors, to produce data that closely resemble real-world driving conditions. In this research, we used the simulator to generate and analyze various aggressive driving scenarios and trajectory data that would be challenging to collect under real road conditions.

**Figure 5:**
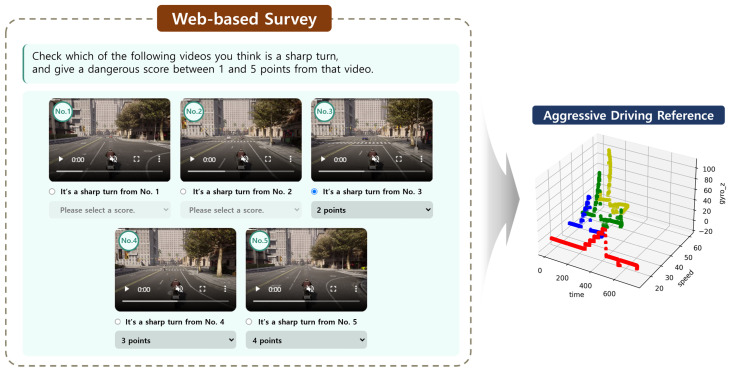
Flowchart for the building of a reference for an inertial sensor. The left section depicts a web-based survey incorporating driving videos from the Carla simulator. Respondents were required to evaluate the degree of aggressiveness in the driving. Subsequently, as shown in the right section, a database of trajectory information from the Carla simulator was recorded.

We conducted surveys with 100 participants to obtain a reference database for aggressive driving situations that respondents collectively identified as dangerous. The left portion of Figure 5 illustrates the web-based system developed to gather this aggressive driving reference database. The survey contained Carla simulator driving videos, and the respondents indicated from which point they considered the driving behavior to be aggressive. These reference data, thus compiled, were loaded into the database as multivariate time series data.

## 3. Two-Wheeled Vehicle Evaluation System

In this section, we delve into the actual attachment-type sensing devices for two-wheeled vehicles, providing an overview of the structure of the two-wheeled vehicle evaluation system developed in this study.

### 3.1. Attachment-Type Aggressive Driving Sensing Device for Two-Wheeled Vehicle

**Figure 6:**
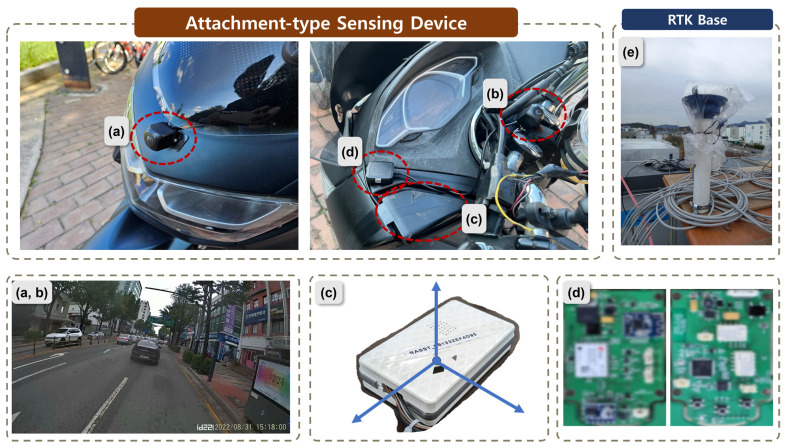
Attachment-type aggressive driving sensing device. (**a**) and (**b**) are cameras and take front and rear images, respectively. (**c**) is an inertial sensor. (**a**) is the front camera, (**b**) is the camera for helmet detection, (**c**) is the inertial sensor, (**d**) is the GPS/GNSS sensor, and blur processing was performed for security. (**e**) is the RTK base.

Figure 6 presents a physical image of the sensing device attached to real two-wheeled vehicles, where (a) and (b) represent the front and rear cameras used to detect image-based risk factors. The front camera (a) is attached to the front part of the two-wheeled vehicle, while the rear camera (b) is mounted at the central position of the handlebars. In (c), the IMU sensor used to detect sensor-based risk factors is shown. The IMU sensor’s data may accumulate noise as the device shakes when the two-wheeled vehicle moves freely. To address this, we integrated an automatic calibration feature into the device and positioned it beneath the two-wheeled vehicle’s glove box or seat to minimize shaking. In, (d) the device used to detect GPS/GNSS-based risk factors is shown, and it communicates location information by connecting to the (e) RTK base station. For security reasons, we applied blurring to the device in (d). These devices are attached to the left and right sides of the two-wheeled vehicle’s dashboard. Power for these devices is supplied through the two-wheeled vehicle’s cigarette lighter socket (12 V) or a USB C-type (5 V) connection.

These attachment-type sensing devices for two-wheeled vehicles communicate with the rider’s mobile phone via a BLE connection, as illustrated in Figure 1. These devices have the following specifications: the CPU is a Rockchip RK3399, equipped with 4GB LPDDR4 memory to support AI detection capabilities. It has 32GB of internal storage and is based on the RK3399 aarch64 LINUX operating system. For ease of attachment, AHD sensors are utilized for the cameras in the sensing devices.

### 3.2. System Overview

#### 3.2.1. Sensing Model

**Figure 7:**
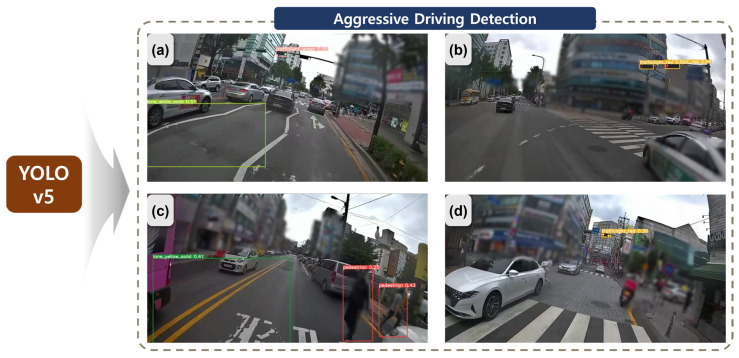
Image-based two-wheeled vehicle dangerous event detection using YOLO v5.

To identify image-based risk factors, this study employed YOLOv5 [41] based on the two-wheeled vehicle training data acquired in Section 2.2.1. Figure 7 provides an example of the results of risk factor detection, where (a) shows the identification of drivable signals from white lane lines and traffic lights, while (b) indicates the detection of stop signals from traffic lights. In (c), the system detects pedestrians in close proximity to the centerline, and (d) shows the detection of stop signals from traffic lights.

After detecting the necessary classes for risk factor identification, a post-processing step is applied to classify events in which two-wheeled vehicles trigger risk factors. The classified information is subsequently transmitted to the evaluation system’s server. In this illustration, certain areas with the potential to expose personal information, such as buildings and people, have been blurred to protect individual privacy.

**Figure 8:**
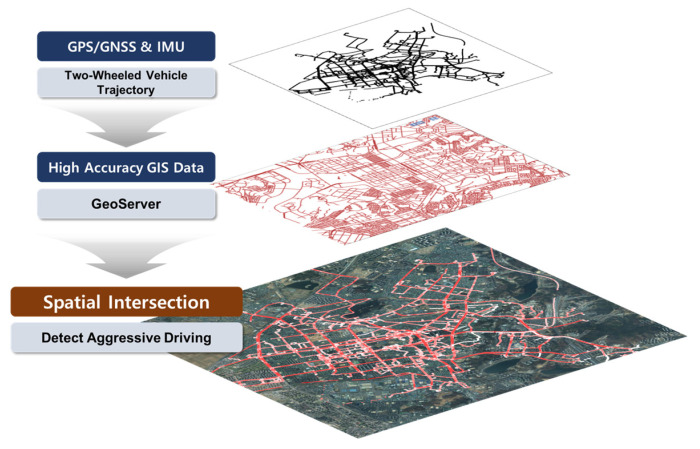
GIS-based aggressive driving detection.

Figure 8 illustrates a schematic representation of risk factor detection based on GIS/GNSS. When data are received from the actual two-wheeled vehicle’s GPS/GNSS and IMU sensors, GeoServer performs spatial operations using the reference data, which are high-precision GIS data, to identify risk factors. For example, in the case of speeding, it was detected by comparing the two-wheeled vehicle’s current speed with the road on which it was traveling in the reference data. Furthermore, given the frequent occurrence of two-wheeled vehicles intruding onto sidewalks in their free driving patterns, this could be distinguished using high-precision GIS reference data that differentiate between roads and sidewalks. As the reference data are managed by GeoServer, there is flexibility in updating the condition values for individual roads. This advantage allows for the continuous updating of various regulations (e.g., school zones, speed limits) for different roads.

**Figure 9:**
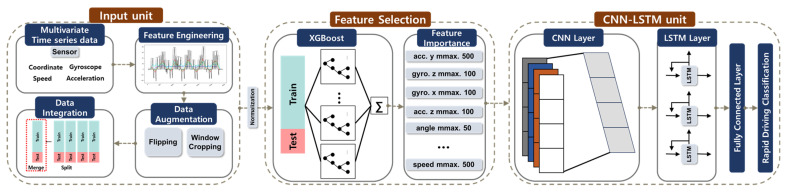
Sensor-based aggressive driving detection.

Figure 9 illustrates the method for the detection of sensor-based risk factors. It shows the sequence of steps to detect aggressive driving based on the multivariate time series data received from the IMU sensor. Unlike image-based and GIS-based items, detecting aggressive driving is considered challenging due to the complex nature of the task. Based on the results of a survey involving 100 participants, it was apparent that distinguishing levels of aggression in the reference data was not a linear process. For this reason, feature engineering techniques and a CNN-LSTM model [42] were used to make this distinction. In Figure 9, the input stage involves preprocessing the multivariate time series data from the reference database, incorporating various characteristics of the training data through feature engineering and data augmentation [43,44]. In the feature selection stage, the top 10 important features were extracted using the XGBoost model [45], effectively serving as a form of dimensionality reduction. Subsequently, a CNN-LSTM model was employed to classify aggressive driving.

#### 3.2.2. Driving Evaluation

Subsequently, a comprehensive evaluation is conducted by considering all 11 identified items. However, it is not reasonable to assume that each of these 11 items should carry the same penalty weight. A survey utilizing these web-based forms targeted 100 participants, achieving a balanced ratio of approximately 1:1:1 among road traffic professionals, two-wheeled vehicle drivers, and non-driving citizens. The balanced ratio was chosen to ensure that perspectives from all relevant stakeholder groups were adequately represented in the survey results. Consequently, in this study, the weights of the items were determined through this web-based survey involving 100 participants (Figure 5). The survey results were analyzed using the analytic hierarchy process (AHP), and Table 3 presents the resulting weights for each risk factor. The AHP involves a two-level hierarchy, and the weights were derived through pairwise comparisons for each category [46,47].

The survey results indicated that the most critical risk factors were identified as helmet non-usage, speeding, and proximity to pedestrians. Helmet non-usage is a high-priority item as it is directly associated with saving lives in the event of accidents, which is reflected in the significant number of violations and accidents involving two-wheeled vehicles, as shown in Figure 2b.

## 4. Experiments/Applications

In this section, we present the results of applying the system developed earlier in a real testbed environment. The testbed was conducted in Republic of Korea, specifically in the cities of Suwon and Seongnam in Gyeonggi Province, from 1 October to 27 October 2023. We recruited ten two-wheeled vehicle riders to participate in the testbed.

**Figure 10:**
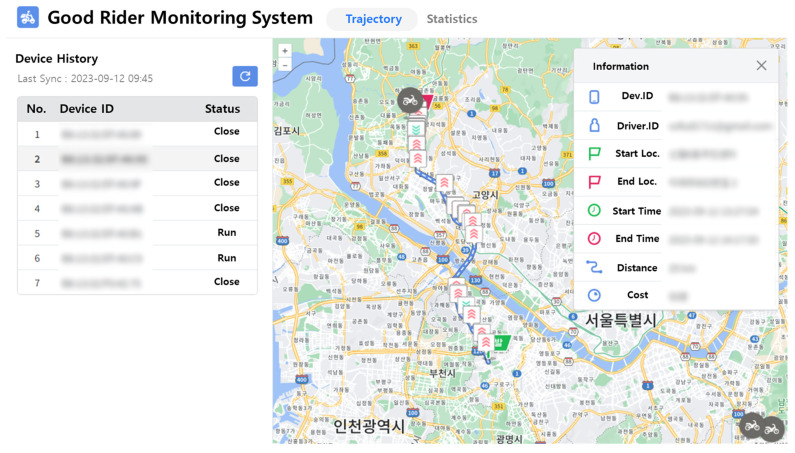
Screenshot of the web interface of the two-wheeled vehicle rider monitoring system. The interface shows the trajectories of real two-wheeled vehicles riders, the risk item violation points, and other information.

In this study, we developed a system to monitor the behavior of two-wheeled vehicles using previously established methods (see Figure 10). This web-based system enables the real-time monitoring of the status and history of two-wheeled vehicle riders. Through the web interface, riders can access information about when and where risk factors were detected. On the left side of the screen, the current riding status of each rider can be observed. Selecting a specific rider displays their riding trajectory on the map, along with details about their driving behavior. The trajectory includes markers indicating the locations of events related to risky driving behaviors. 

**Figure 11:**
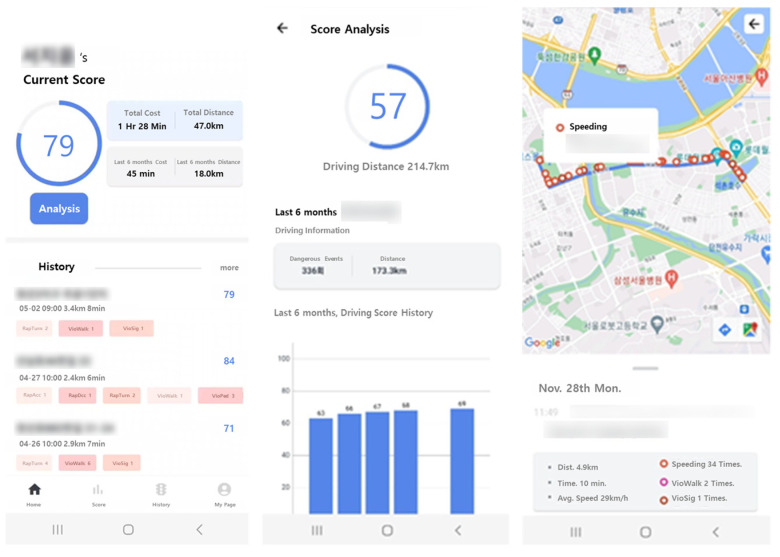
Screenshot of the app interface of the two-wheeled vehicle rider evaluation system.

Figure 11 presents the interface screenshots of the app developed in this study. Through this app, two-wheeled vehicle riders can review their scores for the evaluated trips after they have ended. In the “History” section, riders can track where and to what extent they violated specific items. The evaluation score is quantified by considering factors such as the riding time, the distance traveled, and the frequency of violation items [48]. As a result, individual riders can monitor their driving habits, self-assess, and become aware of when and where they may have triggered particular risk factors.

**Figure 12:**
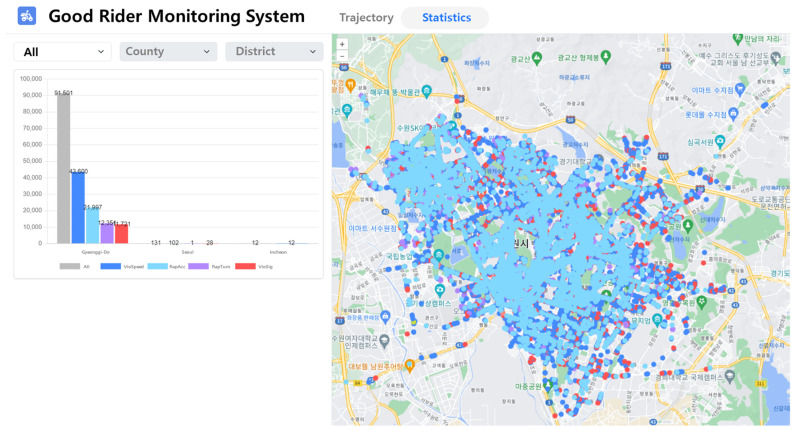
Screenshot of the web interface of the two-wheeled vehicle rider monitoring system. The interface visualizes the location where dangerous driving is detected and presents statistics.

Figure 12 displays a screenshot of the evaluation system monitoring riders’ violations during the testbed period. In the system developed in this study, statistics regarding the locations and timestamps of the detected risk factors for two-wheeled vehicles are available. Furthermore, an assessment is conducted based on the driving trajectory.

Table 4 presents the evaluation results of actual two-wheeled vehicle riders during the testbed period. Due to privacy concerns, data summarization was carried out for only a subset of riders. Although the testbed lasted for one month, the recorded data primarily reflect the pure riding times of riders who perform short-distance deliveries, resulting in shorter recorded times for some of them.

User D achieved a relatively high evaluation score compared to other riders. This rider had a history of regular driving, accounting for 81.269%, with fewer occurrences and a lower intensity of aggressive driving compared to other riders, leading to the higher evaluation score. On the other hand, when comparing User C and User D, their violation patterns were similar, but their evaluation scores varied based on the driving time. This is a result of the score logic, designed to place greater emphasis on recent driving patterns developed by our research team.

However, since this study conducted a real-world test with a limited group of delivery riders who mutually agreed to participate in the project, not all items were detected. In particular, for traffic law violations, the riders continued to operate stably despite prior notification that penalties would not be imposed. In this section, we present the monitoring outcomes for a random selection of four riders. While individual user metrics were examined, it is important to note that our primary focus was not on these specific metrics. Rather, the study aimed to design a fundamental system for the gathering of basic data on two-wheeled vehicle riders.

## 5. Conclusions

In this study, we developed a comprehensive evaluation system to detect risk factors associated with two-wheeled vehicles. We defined eleven specific risk behaviors and proposed various detection methods for each, including image-based, sensor-based, and GIS-based approaches. Dedicated detection devices attached to the vehicles captured the necessary data, which were then evaluated through a web-based system. This system was applied to a testbed area to assess its feasibility and effectiveness. 

However, several limitations need to be addressed. Firstly, as our system remains a prototype, the testbed phase only allowed us to gather data. The practical implementation of detection devices on actual two-wheeled vehicle riders poses a significant challenge. Riders generally hold negative perceptions towards attaching such devices [1,6], necessitating policy-driven incentives. Our ultimate goal extends beyond short-term achievements to contribute to rider safety and overall road safety. To achieve this, we need to encourage voluntary participation from two-wheeled vehicle riders by eliminating evaluation-related penalties within the system. For these reasons, incentive-based approaches were implemented for vehicle safety management.

Additionally, the cost of the detection devices is a significant hurdle, as the prices of the devices used in our testbed ranged from KRW 200,000 to KRW 300,000 per two-wheeled vehicle. Given the characteristics of two-wheeled vehicle riders, the introduction of these devices appears to be a substantial barrier. Therefore, we must lower the cost range of these devices and restructure the key functionalities to address practical considerations.

Furthermore, additional investigation is warranted concerning the relatively low weighted ranking of reverse lane driving and central line violation in the regulatory violation category (Table 3). Addressing these issues in a manner that prioritizes the input of road traffic professionals over a balanced ratio of approximately 1:1:1 among road traffic professionals, two-wheeled vehicle drivers, and non-driving citizens seems more rational.

In conclusion, this study contributes to addressing safety management issues related to two-wheeled vehicles, offering promising avenues for policy applications. Delivery services in Republic of Korea operate as independent businesses without government regulatory oversight, leading to frequent unfair practices among delivery agents during order allocation [49]. There is a lack of legal frameworks to monitor and penalize these unfair behaviors effectively. Due to these regulatory issues, motorcycle delivery riders tend to prioritize faster delivery times to maximize economic gains. Addressing this requires the implementation of long-term measures such as offering mileage incentives, introducing usage-based insurance (UBI), and conducting safety education programs. These initiatives should aim to practically demonstrate the economic benefits of safe driving and remain as future challenges.

## 6. Patents

This section presents a series of patents that embody the technological innovations and methodological advancements developed by our research team and implemented in this study. These patents elucidate the practical applications in the development of advanced systems for the evaluation of driving behavior in two-wheeled vehicles.

US Patent 18/240333 delineates an electronic device designed to detect and assess driving behavior across multiple dimensions. The system employs a variety of sensors and data acquisition methods to monitor driving patterns, including speed, acceleration, deceleration, and directional changes. The analysis of these data yields a comprehensive evaluation of driving behavior, providing insights into both safe and risky driving practices. This multifaceted approach facilitates a more precise understanding of driving dynamics, particularly advantageous for two-wheeled vehicles, where conventional methods may prove inadequate.

US Patent 18/240338 describes an electronic terminal device capable of accumulating historical data to evaluate driving behavior in two-wheeled vehicles. This device records various parameters, including the geographical position, velocity, and duration of travel, which are subsequently utilized to analyze driving patterns over extended temporal periods. Through the compilation and analysis of historical data, the system identifies consistent driving behaviors, detects anomalies, and potentially forecasts future driving trends. This technology has significant implications for traffic safety research, actuarial assessments in the insurance industry, and fleet management operations, where insights into long-term driving behavior are of paramount importance.

This research extends the aforementioned patented principles by incorporating advanced data acquisition and analytical methodologies specifically tailored to motorcycles and scooters. While US Patent 18/240333 establishes a fundamental framework for the detection of multidimensional driving behavior, our study augments this approach by integrating real-time feedback mechanisms and machine learning algorithms optimized for two-wheeled vehicles. Similarly, the emphasis on historical data collection in US Patent 18/240338 is expanded in our research to encompass a broader range of detailed and diverse data points, facilitating a more profound and nuanced analysis of driving behavior over extended periods.

These patents underscore the critical role of technological innovations in enhancing road safety and driving practices. By leveraging these patented methodologies, our re-search endeavors to develop a more robust and comprehensive system for the evaluation and promotion of safe driving behaviors in two-wheeled vehicles.

## Figures and Tables

**Table 1:** Definition of aggressive driving.

	Main Criteria	Sub-Criteria	Results of Two-Wheeled Vehicle Traffic Accident Type Analysis
1	Traffic Violations	Signal Violation	Based on 2017–2021 data for motorcycle accidents in Seoul, Incheon, and Gyeonggi, signal violations account for approximately 23.8% of all accidents. In a survey conducted by the Korea Road Traffic Safety Corporation on signal violation among motorcycles on the roads of Seoul, it constitutes about 48.8% of all violation cases.
2		Reverse Lane Driving	In a survey conducted by the Korea Road Traffic Safety Corporation on wrong-way driving among motorcycles on the roads of Seoul, it constitutes about 3.1% of all violation cases.
3		Central Line Violation	Based on 2017–2021 data for motorcycle accidents in Seoul, Incheon, and Gyeonggi, crossing the centerline accounts for approximately 5% of all accidents. In a survey conducted by the Korea Road Traffic Safety Corporation on crossing the centerline among motorcycles on the roads of Seoul, it constitutes about 5.8% of all violation cases.
4		Speed Violation	In a study of the fatality rates for different types of motorcycle accidents, including speeding, crossing the centerline, and signal violation, speeding exhibits the highest fatality rate at 22.2%.
5		Helmet Non-Usage	According to the 2017–2021 data from the Korean National Police Agency, failure to wear protective headgear, such as helmets, accounts for approximately 50% (around 800,000 cases) of all violations. Among the causes of motorcycle accident-related fatalities, head injuries rank first at a dominant 67.1%.
6	Pedestrian Threat	Proximity to Pedestrians	Based on 2017–2021 data for motorcycle accidents in Seoul, Incheon, and Gyeonggi, cases involving pedestrians as victims constitute about 18% of all accidents (compared to about 48% for passenger vehicles).
7		Riding on Sidewalks	According to the 2017–2021 data from the Korean National Police Agency, cases of motorcycles riding on sidewalks account for approximately 5% of all violations. In a survey conducted by the Korea Road Traffic Safety Corporation on motorcycles riding on sidewalks in Seoul, it constitutes about 11.2% of all violation cases.
8	Reckless Driving	Rapid Acceleration	Motorcycles exhibit significantly higher acceleration per unit weight compared to standard vehicles.
9		Rapid Deceleration	Jackknife effect: In cases of motorcycles or vehicles with a short wheelbase, abrupt braking leads to a shift in weight towards the front, causing the rear wheel to lift and skid.
10		Rapid Turns	According to the Traffic Accident Analysis System (TAAS), over half of the 4035 motorcycle accidents in Seoul in 2021 occurred at intersections.
11		Rapid Lane Changes	The Traffic Accident Analysis System (TAAS) defines lane violations, improper lane usage, overtaking violations, and obstructing straight or right turns as motorcycle accident types.

**Table 2:** Aggressive driving identification methods.

	Main Criteria	Sub-Criteria	Method	References
1	Traffic Violations	Signal violation	Based on Images	[12,13,14]
2		Reverse lane driving	Based on GIS	[15,16]
3		Central line violation	Based on GIS	[17,18,19]
4		Speed violation	Based on GIS	[20,21,22]
5		Helmet non-usage	Based on Images	[23,24,25,26]
6	Pedestrian Threat	Proximity to pedestrians	Based on Images	[27,28,29,30,31]
7		Riding on sidewalks	Based on Images/GIS	[32,33]
8	Reckless Driving	Rapid acceleration	Based on Inertial Sensor	[34,35,36,37,38]
9		Rapid deceleration	Based on Inertial Sensor	
10		Rapid turns	Based on Inertial Sensor	
11		Rapid lane changes	Based on Inertial Sensor	

**Table 3:** Results of AHP-based survey.

	Main Criteria	Sub-Criteria	Weight
1	Traffic Violations	Signal violation	0.082
2		Reverse lane driving	0.060
3		Central line violation	0.096
4		Speed violation	0.124
5		Helmet non-usage	0.158
6	Pedestrian Threat	Proximity to pedestrians	0.122
7		Riding on sidewalks	0.078
8	Reckless Driving	Rapid acceleration	0.098
9		Rapid deceleration	0.098
10		Rapid turns	0.044
11		Rapid lane changes	0.040

**Table 4:** Status of some riders in the testbed.

	Cost (Time) ^1^	Cost (Distance)	Score	Aggressive Driving Event ^2^			
				Normal	Acc.	Dcc.	Turn.
				Ped.	Sig.	Speed.	Helmet.
User A	3 day. 15 h.	1544.330 km	83.237	79.350%	0.460%	0.090%	0.248%
				0.465%	0.002%	19.384%	
User B	12 day. 7 h.	4485.452 km	85.21	78.653%	1.383%	0.233%	0.178%
				0.036%	0.003%	19.513%	0.001%
User C	2 day. 2 h.	1008.265 km	84.44	82.141%	1.245%	0.244%	0.356%
					0.002%	16.013%	
User D	6 day. 2 h.	2215.477 km	99.985	81.269%	0.603%	0.103%	0.083%
						17.953%	

^1^ Minutes have been omitted. ^2^ Acc.: Rapid Acceleration; Dcc.: Rapid Deceleration; Turn.: Rapid Turns; Ped.: Proximity to Pedestrians; Sig.: Signal Violation; Speed.: Speed Violation; Helmet.: Helmet Non-Usage.

## Data Availability

Data sharing is not applicable to this article.

## Abbreviations

The following abbreviations are used in this manuscript:

ACC	Advanced Cruise Control
AHD	Analog High Definition
AHP	Analytic Hierarchy Process
ARAS	Advanced Rider Assistance System
BLE	Bluetooth Low Energy
CNN	Convolution Neural Network
COVID-19	Coronavirus Disease 2019
CPU	Central Processing Unit
DTG	Digital Tachograph
eTAS	Electronic Traffic Assessment Score
GNSS	Global Navigation Satellite System
GPS	Global Positioning System
HMI	Human–Machine Interface
HTTPS	Hypertext Transfer Protocol Secure
IMU	Inertial Measurement Unit
LED	Light Emitting Diode
LSTM	Long Short-Term Memory
OpenAPI	Open Application Programming Interface
RPM	Revolutions Per Minute
RTK	Real-Time Kinematic
TAAS	Traffic Accident Analysis System
UBI	Usage-Based Insurance
WFS	Web Feature Service
WMS	Web Map Service
XGBoost	Extreme Gradient Boosting
